# Spatial transcriptomics reveal heterogeneous cell‒cell interactions among brain regions in cuprizone model consistent with multiple sclerosis lesions

**DOI:** 10.3389/fbinf.2026.1832826

**Published:** 2026-08-06

**Authors:** Hui-Hsin Tsai, Sarbottam Piya, Jing Wang, Jing Zhu, Wenxing Hu, Andrew R. Gehrke, Shaolong Cao, Amanda J. Guise, Su Jing Chan, Mark Sheehan, Jenhwa Chu, Zhengyu Ouyang, Matthew Ryals, Michelle Lee, Wanli Wang, Edward Zhao, Patrick Cullen, Ravi Challa, Eric Marshall, Wanyong Zeng, Yea Jin Kaeser-Woo, Chris Ehrenfels, Luke Jandreski, Helen McLaughlin, Thomas M. Carlile, Jake Gagnon, Taylor L. Reynolds, Mingyao Li, Kejie Li, Baohong Zhang

**Affiliations:** 1 Biogen Inc., Cambridge, MA, United States; 2 Data Science, BioInfoRx Inc., Madison, WI, United States; 3 PharmaLex Inc., Conshohocken, PA, United States; 4 Department of Biostatistics, Epidemiology and Informatics, Perelman School of Medicine, University of Pennsylvania, Philadelphia, PA, United States

**Keywords:** cell-cell interaction, cuprizone model, demyelination, multiple sclerosis, oligodendrocytes, remyelination, snRNA-seq, spatial transcriptomics (ST)

## Abstract

The cuprizone (CPZ) model is widely used for modeling demyelination in multiple sclerosis (MS) and for testing potential remyelination therapies. To better understand the underlying pathology of the CPZ model and evaluate its translatability, we integrated single-cell and spatial transcriptomics (ST) to investigate spatial cellular and molecular interactions during de- and remyelination in multiple brain regions. ST revealed global demyelination and neuroinflammation in the brain beyond the corpus callosum (CC), with region-specific differences. We identified oligodendroglia and microglia as two major cell types with significant transcriptomic changes in the model. CPZ-associated subclusters of oligodendroglia (marker genes *Arap2*, *Dock10*, *Tenm4*, *Pex5l* and *Dock1*) and microglia (marker genes *ApoE*, *Axl*, *Cd9* and *Lpl*) were mapped to the CC by ST. During remyelination, while mature oligodendrocytes (MOL) nearly reversed their phenotype back to the control state, microglia remained associated with the demyelination phenotype. Ligand‒receptor (LR) pairing analyses predicted growth factor and phagocytic pathway enrichment during demyelination, which is consistent with changes in MS lesions, and microglia were predicted to be the major sender cells. LR pairing also predicted a high likelihood of interaction between oligodendroglia and microglia, and a novel interaction between MOL and oligodendrocyte precursor cells (OPC), underscoring their roles during de- and remyelination. Finally, astrocytes in the CPZ model had the greatest preservation of disease-associated modules in MS lesions, while MOL, OPC, and microglia showed moderate to low preservation, which overall suggests that the CPZ model has moderate translatability to chronically active MS lesions.

## Introduction

Multiple sclerosis (MS) is an inflammatory demyelinating disease of the central nervous system (CNS) with pathological hallmarks of gray and white matter demyelination, neurodegeneration, inflammation, and glial reactions (Lassmann, 2018; Reich et al., 2018). A genome-wide association study (GWAS) previously highlighted the role of peripheral immune cells in MS disease risk (International Multiple Sclerosis Genetics Consortium, 2019). Indeed, approved MS therapies targeting peripheral inflammation have shown high efficacy in dampening disease activities. However, despite well-controlled acute inflammation, disease progression can continue unabated, resulting in increased disability that currently lacks effective treatments (Ciccarelli et al., 2024). A recent GWAS revealed a locus associated with MS progression (International Multiple Sclerosis Genetics Consortium MultipleMS Consortium, 2023). Notably, the locus does not overlap with previously identified immunological susceptibility genes. The nearest gene to the locus, dysf, encodes DYSFERLIN, a protein specifically expressed by oligodendrocytes and neurons, underscoring CNS tissue involvement in MS disease progression. Therefore, it is critical to understand the mechanisms underlying the progressive biology at the cellular and molecular levels in the CNS and pave the paths for identifying potential drug targets to treat MS disease progression.

The cuprizone (CPZ) model is widely used to model the MS demyelination process and/or test potential new therapies for remyelination (Vega-Riquer et al., 2019). CPZ is a copper chelator that inhibits mitochondrial functions, causing the selective degeneration of mature oligodendrocytes (MOLs) (Kipp, 2024). The model has a stereotypical spatial-temporal demyelination-remyelination course, and the corpus callosum (CC), which is the most studied brain region in the CPZ model, shows a highly predictable demyelination pattern. During CPZ intoxication, demyelination and remyelination events may coexist at the same time as the lesion evolves until peak demyelination is reached. This feature has been considered a strength of the model to mimic early-stage MS lesions when damage and repair can occur concurrently (Barkhof et al., 2003; Ransohoff, 2012). The CNS spatial phenotypes in the CPZ model, such as demyelination, neurodegeneration, and gliosis, are traditionally characterized by histological staining, immunohistochemistry (IHC), and electron microscopy (Stidworthy et al., 2003; Gudi et al., 2014; Gudi et al., 2017). While histopathological examination preserves findings relating to anatomical architecture, the disadvantage is that only a few selected markers can be examined, and the overall cellular and molecular response is not fully captured.

In recent years, both the CPZ model and MS have been investigated by omics-based efforts to better understand the underlying pathology of neuroinflammation and demyelination (Hou et al., 2023; Alsema et al., 2024; Lerma-Martin et al., 2024). However, these studies usually focused on one type of omics and investigated either the CPZ model or MS with the technology. In our current study, we integrated multiple types of omics data with several methodologies to analyze the CPZ model and evaluate the CPZ model and MS datasets together when appropriate. We aimed to unbiasedly unwrap cellular and molecular changes during de- and remyelination in the CPZ model by employing spatial transcriptomic (ST) technology (Liu et al., 2021; Rao et al., 2021) to preserve the anatomical context. We have resolved spatial gene expression in the model by integrating the ST data with the single-nucleus RNA-seq (snRNA-seq) reference in the same study. We found that demyelination and neuroinflammation were prominent and widespread in the CPZ brain with brain region-specific gene expression changes. Ligand‒receptor (LR) pairing analyses revealed an enrichment of phagocytic and growth factor pathways in the CPZ group from both the snRNA-seq and ST data. Finally, we compared the snRNA-seq data from our study with a human MS dataset (Absinta et al., 2021) and found a moderate level of preservation of MS-associated disease modules in the CPZ model. In the future, the methodology developed in this study can be applied to human MS samples to map expression patterns and changes in the heterogeneous lesion environment (Kuhlmann et al., 2017).

## Results

**FIGURE 1:**
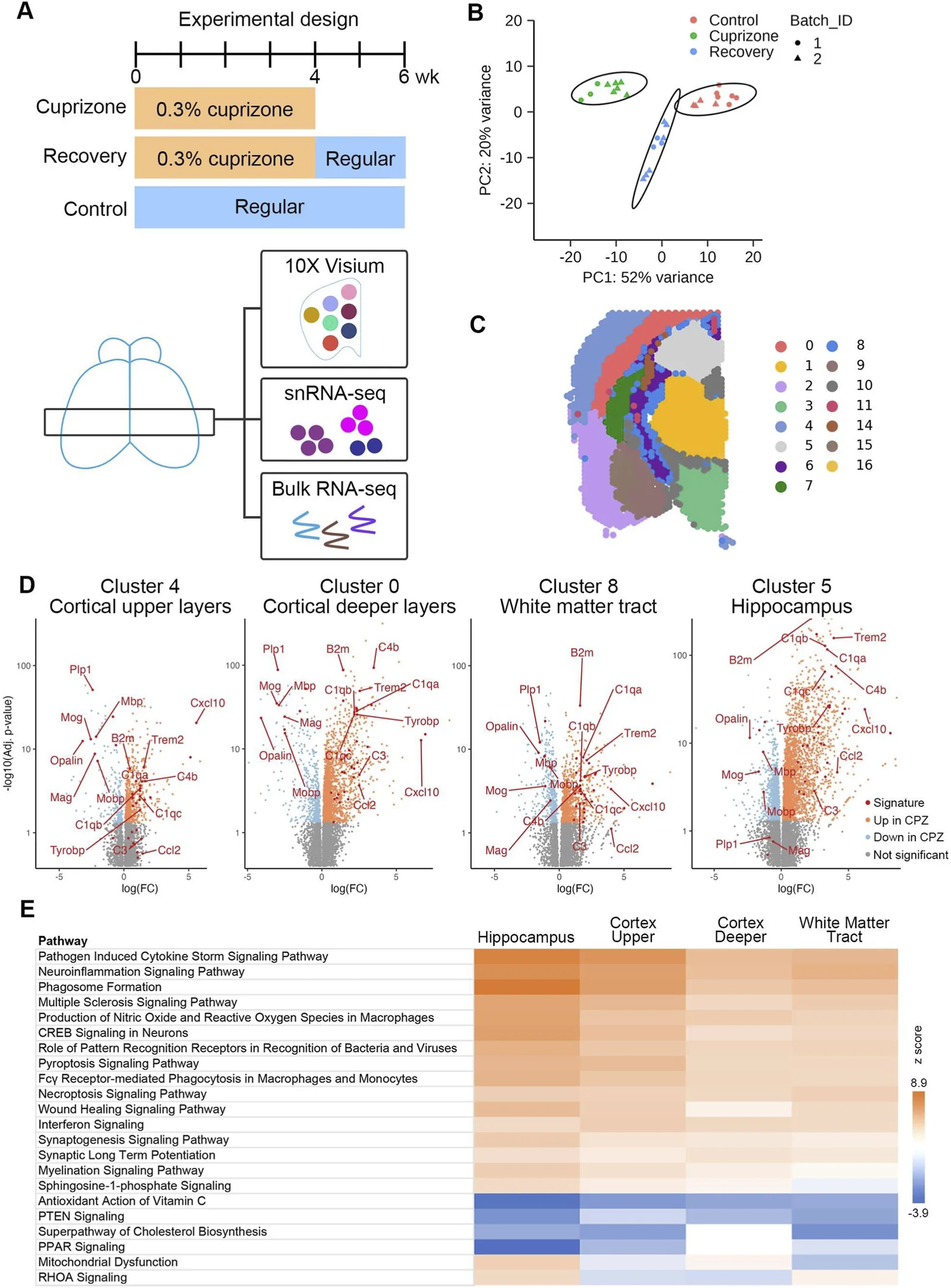
Widespread demyelination and neuroinflammation occur along the dorsal-ventral axis of the CPZ brain. **(A)** CPZ model experimental design. Three experimental groups (control, CPZ, and recovery) were collected in this study. **(B)** Spatial transcriptomic data from three experimental groups were distinctly separated on the PCA plot after pseudobulking. PCA plot of spot-level pseudobulk across biological replicates from the ST data, with each point representing a biological replicate. The point color represents the experimental conditions, and the point shape represents the batch. **(C)** The 17 clusters of ST spots identified by the SpaGCN algorithm strongly resembled the reference anatomical annotations (Allen Brain Atlas mouse reference atlas: https://mouse.brain-map.org/). **(D)** Volcano plots of differentially expressed genes (DEGs) between CPZ and control conditions in cluster 4 (cortical upper layers), cluster 0 (cortical deeper layers), cluster 8 (white matter tract) and cluster 5 (hippocampus) are labeled with a list of curated MS signature genes. The negative log adjusted p values (y-axis) are presented on a log scale. **(E)** The majority of pathways showed congruous directionality among clusters, while some showed incongruous directionality. DEGs in each cluster were analyzed by IPA core analysis for pathway discovery and subsequently subjected to comparison analysis. A total of 13 samples were collected, including 5 control samples, 4 CPZ samples, and 4 recovery samples. Bulk RNA-seq data were generated for all 13 samples. Due to budget constraints, scRNA-seq and ST data were obtained for 9 of the 13 samples, with 3 samples from each group.

We collected brain tissues (approximately bregma −1.5 mm) from three experimental groups of mice (∼10 weeks old): (1) the cuprizone group (CPZ), in which mice were fed with 0.3% CPZ for 4 weeks to induce peak demyelination, (2) the recovery group (RCV), in which mice received 4 weeks of CPZ followed by 2 weeks of normal chow to allow for spontaneous remyelination, and (3) the control group (CTL), which received normal chow for 6 weeks. Three different sequencing platforms—snRNA-seq, bulk RNA-seq, and 10X Visium spatial gene expression profiling (Figure 1A, Methods)—were used to analyze the gene expression patterns.

### ST reveal global demyelination and region-specific transcriptomic changes induced by CPZ

We first compared the Visium spatial maps of *Mbp, Cd68*, and *Gfap* genes at the spot level with IHC staining of proteins and showed that the expression patterns were comparable between the Visium and IHC data (Supplementary Figure S1). These genes were chosen based on their well-established expression patterns during de- and remyelination in the CPZ model. The ST dataset contained a total of 57,217 spots (average = 2,043) from all the sections analyzed, with an average of 75,141 reads per spot and a median of 4,157 genes per spot. To look at the overall structure of the ST dataset, we performed unsupervised spot-level pseudobulk principal component analysis (PCA) by aggregating spot counts for each gene across all spots within each section to form a gene-by-section count matrix. Our results showed that the three experimental groups were distinctly separated along PC1 (Figure 1B). We also observed concordance of cell abundance estimates in the same region between animals after ST cell type deconvolution (Supplementary Figure S2), which further suggested that the separation shown by PCA was driven by experimental groups.

We performed unbiased clustering of the ST spots using the SpaGCN package (Hu et al., 2021) and identified 17 spatial clusters closely resembling the reference anatomical regions (http://www.brain-map.org) (Figure 1C). The identification of clusters was based on spatially variable genes enriched in anatomical domains and enabled by coherent expression and histology mapping. We identified 484, 1495, 1086 and 2762 differentially expressed genes (DEGs) between CPZ and CTL groups in Cluster 4 (occupying cortical upper layers, roughly layers I-III), Cluster 0 (occupying cortical deeper layers, roughly layers IV-VI), Cluster 8 (roughly occupying white matter tract), and Cluster 5 (roughly occupying hippocampus), respectively. Cluster 5 (hippocampus) had the greatest number of DEGs and highest fold changes among the 4 clusters (Figure 1D). We curated a list of genes that represent MS gene signature (Method) (Jäkel et al., 2019; Absinta et al., 2021) and found the upregulation of proinflammatory genes (*B2m, Cxcl10*, *C4b*, and *Trem2*) and the downregulation of myelin-related genes (*Mbp* and *Mog*) in all 4 clusters (Figure 1D). While the CC has been viewed as the major anatomical location for evaluating demyelination and remyelination in the CPZ model (Xie, 2010; Praet et al., 2014), our analysis showed that inflammatory and demyelination gene signatures were prominent and widespread in various brain regions. Further analysis of biological pathways revealed that neuroinflammation and cell death pathways were prominently upregulated in all 4 clusters/regions. PTEN signaling and the superpathway of cholesterol biosynthesis were downregulated in all 4 clusters, and they have both been linked to myelin integrity and demyelination (Harrington et al., 2010; Itoh et al., 2017) (Figure 1E).

Clusters 4 and 0 occupied different layers of the cortex. Higher number of genes from the MS gene signature reached statistical significances in Cluster 0 compared with Cluster 4, suggesting a higher inflammatory state in deeper cortical layers. This observation parallels a previous finding that the upper cortical layers remyelinated more robustly compared with the deeper layers in the CPZ model, and the difference in the repair capacity was attributed to a hypothesized increase in inflammatory activity, myelin debris, and gliosis in the deeper layers (Orthmann-Murphy et al., 2020).

### Oligodendroglia and microglia are the major cell types associated with the CPZ model

**FIGURE 2:**
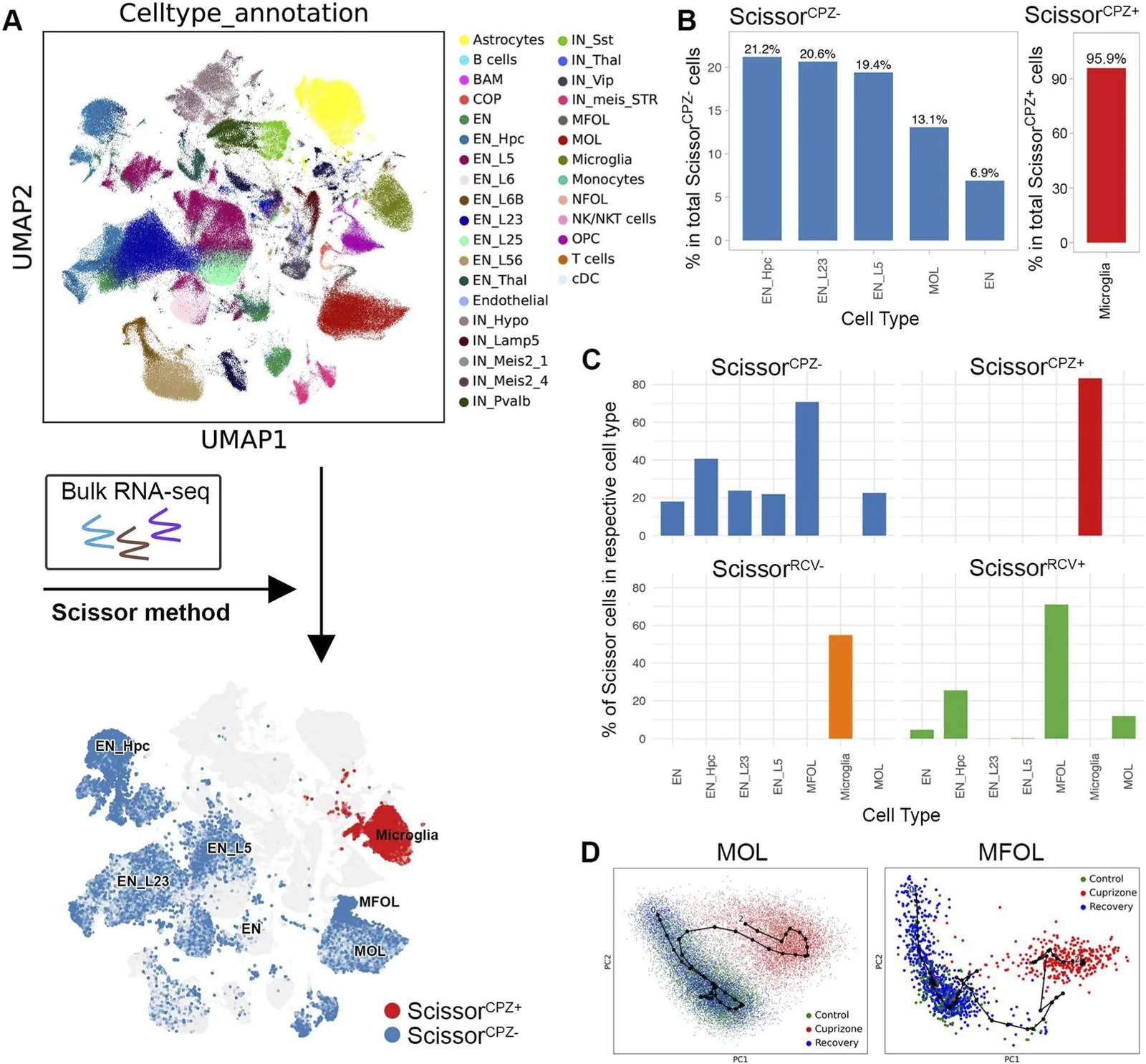
Identification of CPZ-associated cell types by integrating bulk and snRNA-Seq data with Scissor. **(A)** Single-cell UMAP projection of the mouse brain snRNA-Seq data colored by cell annotation. By integrating bulk RNA-seq data, blue Scissor^CPZ−^ and red Scissor^CPZ+^ were identified. **(B)** Bar plots demonstrating a breakdown of the cell type compositions within the total of Scissor^CPZ−^ or Scissor^CPZ+^ cells. **(C)** Bar plots showing a detailed breakdown of the percentage of Scissor cells in each cell type. **(D)** Pseudotime analysis of snRNA-Seq data for MOL and MFOL showed that their CPZ phenotypes were distinct from those of the control and RCV phenotypes.

To resolve cell types at each ST spot, we dissected samples from dorsal brain regions, including the cortex, CC, and hippocampus, to perform snRNA-seq. We obtained a total of 294,447 nuclei with a mean of 3,392 detected genes per nucleus after filtering out low-quality cells and potential doublets and removing ambient RNAs. By transferring cell labels from publicly available datasets, we identified a total of 32 annotated cell types and validated cell purity (Figure 2A; Supplementary Figure S8; Supplementary Table S3) (Marques et al., 2016; Kleshchevnikov et al., 2022; Scheyltjens et al., 2022).

To identify cell types that are associated with treatment conditions, we took two complementary approaches. First, we integrated both bulk and single-cell data using the Scissor package to unbiasedly identify cell populations associated with treatment conditions by asking which single-cell expression profiles best explain the phenotype differences based on the bulk data labels of CTL, CPZ, and RCV (Sun et al., 2022). In the CPZ and CTL contrast, we found that 2.99% of cells (8,802 cells among 294,447 nuclei) were positively associated with the CPZ phenotype (Scissor^CPZ+^), 14.9% of cells (43,891 cells) were associated with the CTL phenotype (Scissor^CPZ−^), and the remaining cells were considered background cells (Figures 2B,C). Among Scissor^CPZ+^ cells, 95.9% were microglia and within the microglial population, 83.3% were classified as Scissor^CPZ+^. This result suggests that microglia are highly correlated with CPZ phenotype, if not the driving force of the phenotype. On the other hand, the cell types were more diverse among Scissor^CPZ−^ cell. We found 13.1% were MOL, 2.14% were MFOL (myelin-forming oligodendrocytes), 21.2% were EN_Hpc, 20.6% were EN_L23, 19.4% were EN_L5 and 6.9% were EN. Furthermore, 70.8% of MFOL, 40.6% of EN_Hpc, 22% of MOL, 23.8% of EN_L23, 21.9% of EN_L5 and 18% of EN were classified as Scissor^CPZ−^ within their respective cell type populations (Figure 2C). In the CPZ and RCV contrast, 4.13% of the cells (12,182 cells) were positively associated with the RCV phenotype (Scissor^RCV+^ cells), and 1.95% of the cells (5,729 cells) were associated with CPZ phenotype (Scissor^RCV−^ cells). The Scissor^RCV+^ cells comprised 25% MOL, 48% EN_Hpc, and 7.8% MFOL, and the Scissor^RCV−^ cells comprised 96.6% microglia. This result suggests that oligodendroglial populations were closely associated with the RCV phenotype. While some microglia had adopted the RCV phenotype, there was still a considerable microglia population that remained associated with the CPZ phenotype. Among the Scissor cell types, microglia had the greatest number of DEGs, and neurons generally had lower numbers of DEGs (Supplementary Figure S3).

**FIGURE 3:**
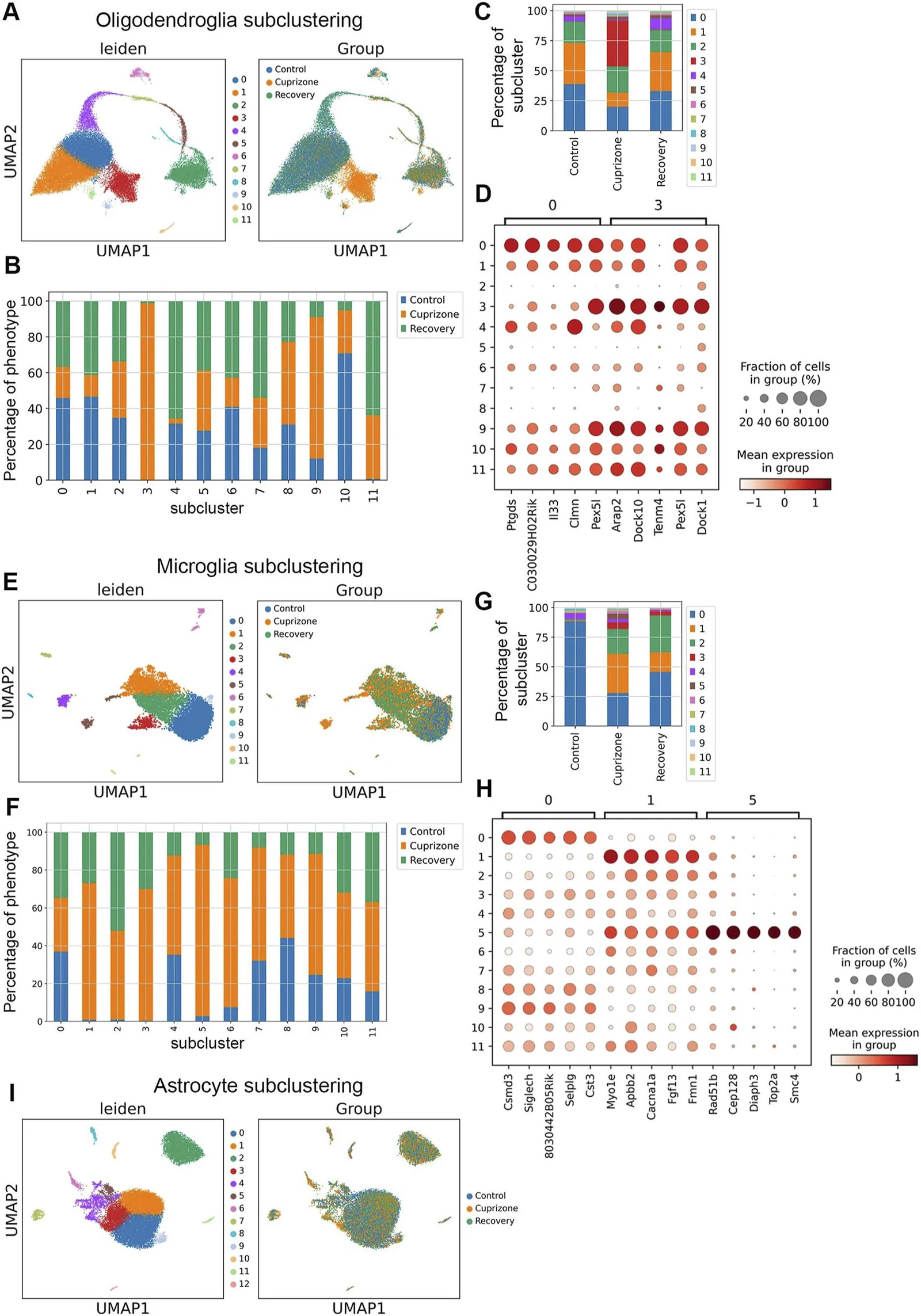
Identification of CPZ-associated oligodendroglia and microglial subclusters. **(A)** The UMAP of oligodendroglia is colored according to subclusters and are separated by phenotype. **(B)** Stacked bar plot of oligodendroglial subclusters according to treatment phenotype. **(C)** Stacked bar plot of treatment phenotypes according to the percentages of oligodendroglial subclusters. **(D)** Dot plot of the top 5 marker genes specific to subcluster 0 and subcluster 3 (CPZ-associated subclusters) of oligodendroglia. **(E)** The UMAP of microglia is colored according to subcluster and are separated by phenotype. **(F)** Stacked bar plot of microglial subclusters according to treatment phenotype. **(G)** Stacked bar plot of treatment phenotypes colored according to the percentages of microglial subclusters. **(H)** Dot plot showing the top 5 marker genes specific to subcluster 0 (homeostatic subcluster) and subclusters 1 and 5 (CPZ-associated subclusters) of microglia. **(I)** UMAP of astrocytes colored according to subcluster did not show separation according to phenotype.

Secondly, we performed differential expression analysis using NEBULA across all annotated cell types in our snRNA-seq dataset (22 cell types passing quality filters) for all three contrasts (CPZ vs. Control, Recovery vs. Control, CPZ vs. Recovery), using FDR <0.05 and |log2FC| > 0.25 as significance thresholds (Supplementary Figure S7; Supplementary Table S3). In the CPZ vs. Control contrast, MFOL showed the highest DEG count, followed by MOL and microglia despite a small population of MFOL (n = 1,331). In the CPZ vs. RCV contrast, MFOL and MOL had highest numbers of changes (2,085 and 1,125 DEGs, respectively), while microglia showed a more moderate change (418 DEGs), consistent with our finding that microglia maintained a sustained demyelination-associated phenotype during recovery (Figures 2C, 3G).

Since CPZ selectively targets the vulnerable oligodendrocyte population, it is reassuring to see them identified by both methods. Microglial involvement in the model is also consistent with previous publications using histology or omics methods (Gudi et al., 2009; Gudi et al., 2014; Hou et al., 2023). The results of Scissor further suggests that microglia exhibited a sustained demyelination phenotype, while oligodendroglia exhibited a phenotype closer to that of the CTL group after 2 weeks of remyelination. The similarity of oligodendroglia between the RCV and CTL phenotypes was further supported by pseudotime analysis. That is, the CPZ phenotype was distant from the RCV and CTL phenotypes, while the RCV and CTL phenotypes were indistinguishable from each other (Figure 2D). While we recognize some technical limitations of Scissor, the convergence of phenotype association by Scissor and the direct differential expression analysis by NEBULA provides orthogonal support for our conclusion that oligodendroglia and microglia are the principal cell types affected in the CPZ model.

### Identification of oligodendroglial and microglial subclusters associated with CPZ phenotype

Following the Scissor finding, we focused on oligodendroglia and microglia in the snRNA-seq dataset. We identified 12 subclusters from oligodendroglia, including subclusters 0, 1, 3, 4, 6, 9, 10, and 11 from MOLs; subclusters 3, 4, and 6 from MFOLs; subclusters 5 and 7 from NFOLs (newly formed oligodendrocytes); subcluster 5 from COPs (differentiation-committed oligodendrocyte precursors); and subclusters 2 and 8 from OPCs (oligodendrocyte precursors) (Figure 3A) using the Leiden clustering algorithm (see Material and Method). Based on T-test, the proportion of cells in subcluster 3 increased significantly in the CPZ condition (CPZ vs. CTL, p value = 6.223e-10) and decreased significantly in the RCV condition (RCV vs. CPZ, p value = 5.861e-10) (Figures 3B,C). This result supports that subcluster 3 is exclusively associated with the CPZ condition and expresses the marker genes *Arap2, Dock10, Tenm4, Pex5l* and *Dock1* (Figure 3D). Most of these genes are involved in small GTPase signaling and cytoskeleton remodeling (Thurnherr et al., 2006), which suggests extensive oligodendrocyte structural remodeling associated with the CPZ condition. Mutations of some of these marker genes have been linked with dysregulation of myelination (Hor et al., 2015; Doan and Monk, 2025). On the other hand, subclusters 0, 1, and 4 showed a marked reduction in proportion in the CPZ condition (CPZ vs. CTL, p value = 0.006, 0.0004, and 0.0475, respectively). The proportions of these 3 subclusters increased in the RCV condition, suggesting a phenotype reversal, i.e., remyelination (RCV vs. CPZ, p value = 0.03, 0.001, and 0.002, respectively) (Figure 3C).

We also identified 12 subclusters from microglia (Figures 3E–H). More than 88% of the CTL microglia were assigned to subcluster 0, which expressed homeostatic genes, such as *Tmem119, P2ry12, Tgfbr1*, and *Siglech*. Microglia expanded substantially during demyelination, and the proportions of microglia in the CTL, CPZ and RCV groups were 19%, 46.5% and 34.5%, respectively. CPZ-associated subcluster 1 showed high expression of the *ApoE, Axl, Cd9* and *Lpl* genes, which have been implicated as neurodegenerative disease-associated genes (Keren-Shaul et al., 2017). The proportion of subclusters 1 and 5 exhibited a significant increase during demyelination (CPZ vs. CTL, p value = 1.567e-08 and 0.029, respectively), followed by a significant decrease during remyelination (RCV vs. CPZ, p value = 0.009 and 0.035, respectively) (Figure 3G). Unlike those in oligodendroglia, the changes in the proportions of microglial subclusters from the CPZ to the RCV condition are far from normalization compared to those in the CTL condition. Surprisingly, we did not find any subclusters of astrocytes associated with the CPZ phenotype (Figure 3I).

### CellChat identified phagocytosis, growth factor, and chemokine pathways as novel cell-cell interactions from the CPZ condition in the snRNA-seq dataset

We analyzed the snRNA-seq dataset by CellChat (Jin et al., 2021) to uncover novel enriched ligand-receptor (LR) pairs. We were particularly interested in “novel” LR pairs that appeared and were significant in the CPZ condition but either did not exist or were not significant in the CTL condition.

**FIGURE 4:**
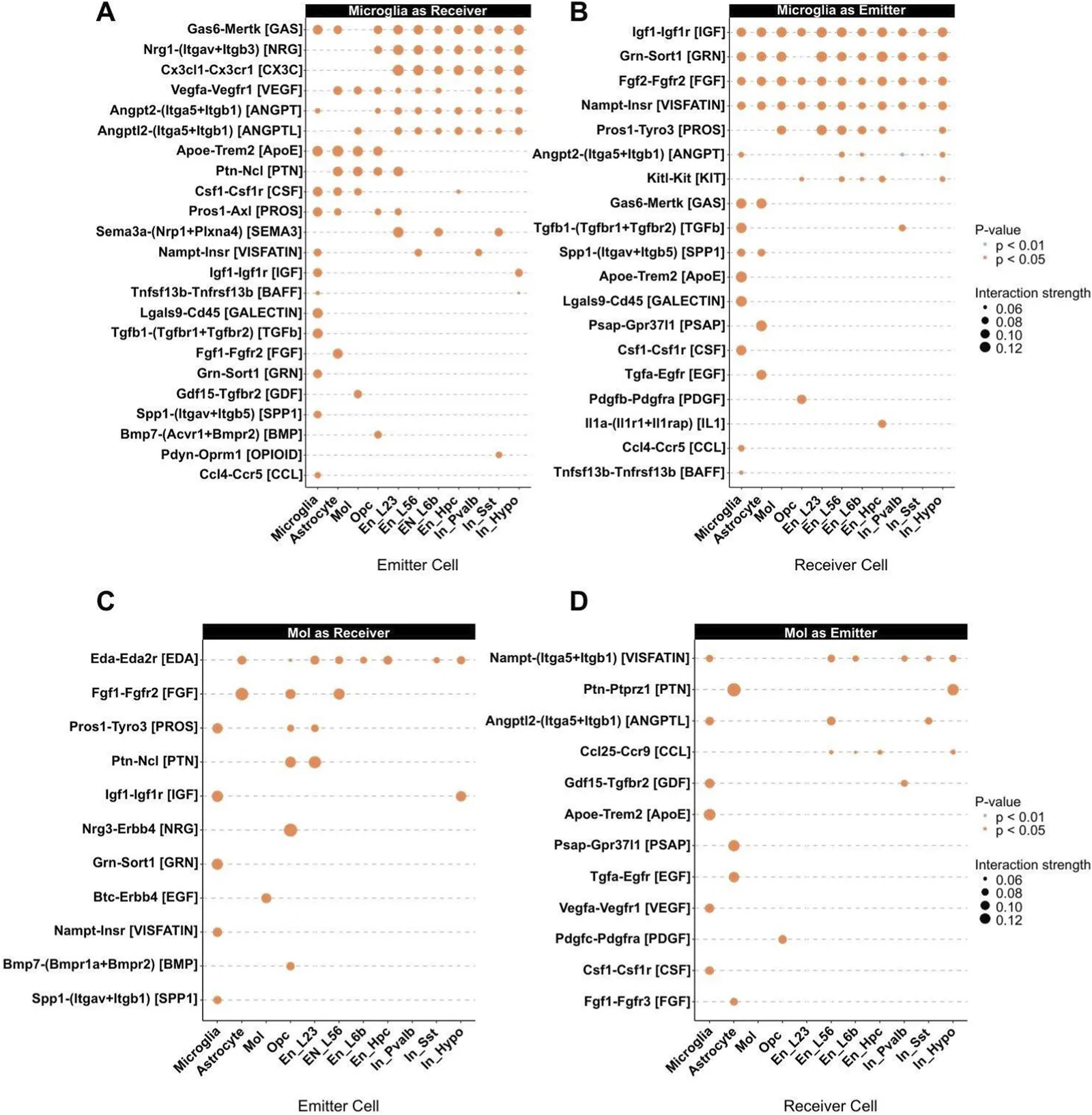
Novel LR pairs under the CPZ condition predicted from the single-cell dataset by CellChat. **(A)** CellChat analysis of the snRNA-seq data predicted novel CPZ LR pairs with microglia as the receiver. The associated CellChat pathway information is listed in brackets beside the LR name along the vertical axis and sender cell types along the horizontal axis. The point size represents the CellChat interaction strength. The point color indicates the significance cutoff. **(B)** Novel LR pairs with microglia as sender cells. **(C)** Novel LR pairs with MOL as a receiver cell. **(D)** Novel LR pairs with MOL as a sender cell.

We identified enhanced phagocytosis, growth factor, and chemokine pathways in microglia, either as receiver or emitter cells under the CPZ condition (Figures 4A,B). When microglia were analyzed as receiver cells, they paired with multiple emitter cell types with significant novel LR pairs, such as GAS (Gas6-Mertk), NRG (Nrg1-(Itgav + Itgb3)), CX3C (Cx3cl1-Cx3cr1), VEGF (Vegfa-Vegfr1), ANGPT (Angpt2-(Itga5 + Itgb1)), and ANGPTL (Angptl2-(Itga5 + Itgb1)) (Figure 4A). The Gas6-Mertk pathway is considered an anti-inflammatory pathway and has been shown to be critical for modulating microglial phagocytic activity to remove myelin debris, therefore promoting remyelination in the CPZ model (Shen et al., 2021). Interestingly, pathways involved in blood vessel remodeling, such as the VEGF and ANGPT pathways, were predicted to be present in microglia (Proescholdt et al., 2002; Thurston and Daly, 2012). Increasing evidence has suggested that interactions between microglia and blood vessels could play roles in MS and other diseases (Davalos et al., 2012; Zhao et al., 2018). Other novel LR pairs in the CPZ condition included FGF (Fgf1-Fgfr2, astrocytes to microglia), GDN (Gdf15-Tgfbr2, MOL to microglia), and BMP (Bmp7-(Acvr1 + Bmpr2), OPC to microglia) pairs. Growth differentiation factor 15 (GDF15) is a neurotrophic factor of the TGFβ superfamily, and elevated levels of GDF15 in serum are associated with MS disease stability and are presumed to be anti-inflammatory (Amstad et al., 2020). *Gdf15* was recently identified as a marker of demyelination-associated oligodendrocytes and was specific to the CPZ model but not the 5XFAD model of Alzheimer’s disease (Hou et al., 2023). Here, we further predicted that *Gdf15* expression in MOLs may exert its function through Tgfbr2 on microglia.

When microglia were analyzed as emitter cells, interactions between IGF (Igf1-Igf1r), GRN (Grn-Sort1), FGF (Fgf2-Fgfr2), VISFATIN (Nampt-Insr), and PROS (Pros1-Tyro3) were predicted in multiple receiver cell types (Figure 4B). PSAP (Psap-Gpr37l1, microglia to astrocytes), EGF (Tgfa-Egfr, microglia to astrocytes), PDGF (Pdgfb-Pdgfra, microglia to OPC), and IL1 (Il1a-(Il1r1 + Il1rap), microglia to EN-Hpc) pathway interactions were predicted between microglia and selective cell types. Finally, TGFb (Tgfb1-(Tgfbr1 + Tgfbr2)), ApoE (ApoE-Trem2) and Csf1-Csf1r (CSF) were identified as pathway interactions between microglia. Among these pathways, most of them have been demonstrated to be neuroprotective, while the CSF1-CSF1R interaction is considered proinflammatory. For example, the IGF pathway could play a role in decreasing the severity of neuroinflammation (Ivan et al., 2023), and Gpr37l1 expression in astrocytes modulates neuronal N-methyl-D-aspartate (NMDA) receptor activation during ischemia to exert protective effects (Jolly et al., 2018). So far, LR analysis by CellChat revealed that microglia interacting pairs may provide protective functions in addition to proinflammatory functions under CPZ conditions.

Most of the LR pairs identified from MOL were involved in growth factor signaling pathways (Figures 4C,D), such as the FGF, EGF, IGF, and VEGF pathways, regardless of whether the MOL was a receiver or emitter cell type. Interestingly, our analysis also revealed MOL and OPC interactions under the CPZ condition. When MOLs were analyzed as receiver cells, they received FGF, PTN and NRG signaling from OPCs, whereas OPCs received PDGF signaling from MOLs (Pdgfc-Pdgfra, OPC to MOL) when the MOLs were analyzed as emitter cells. To our knowledge, the cell-cell interaction between MOLs and OPCs is novel and could be important for understanding the repair capacity and/or maintenance of homeostasis of oligodendrocytes in diseases.

The CellChat analysis revealed that while microglia have a high likelihood of interacting with other microglia and astrocytes, they also interacted with MOLs and OPCs. Conversely, MOLs have a high likelihood of interacting with microglia. The data so far suggests that not only could oligodendroglia and microglia play major roles during de- and remyelination, but they may also interact directly with each other throughout the process (Baaklini et al., 2023).

### ST maps CPZ-associated cell subclusters to CC

**FIGURE 5:**
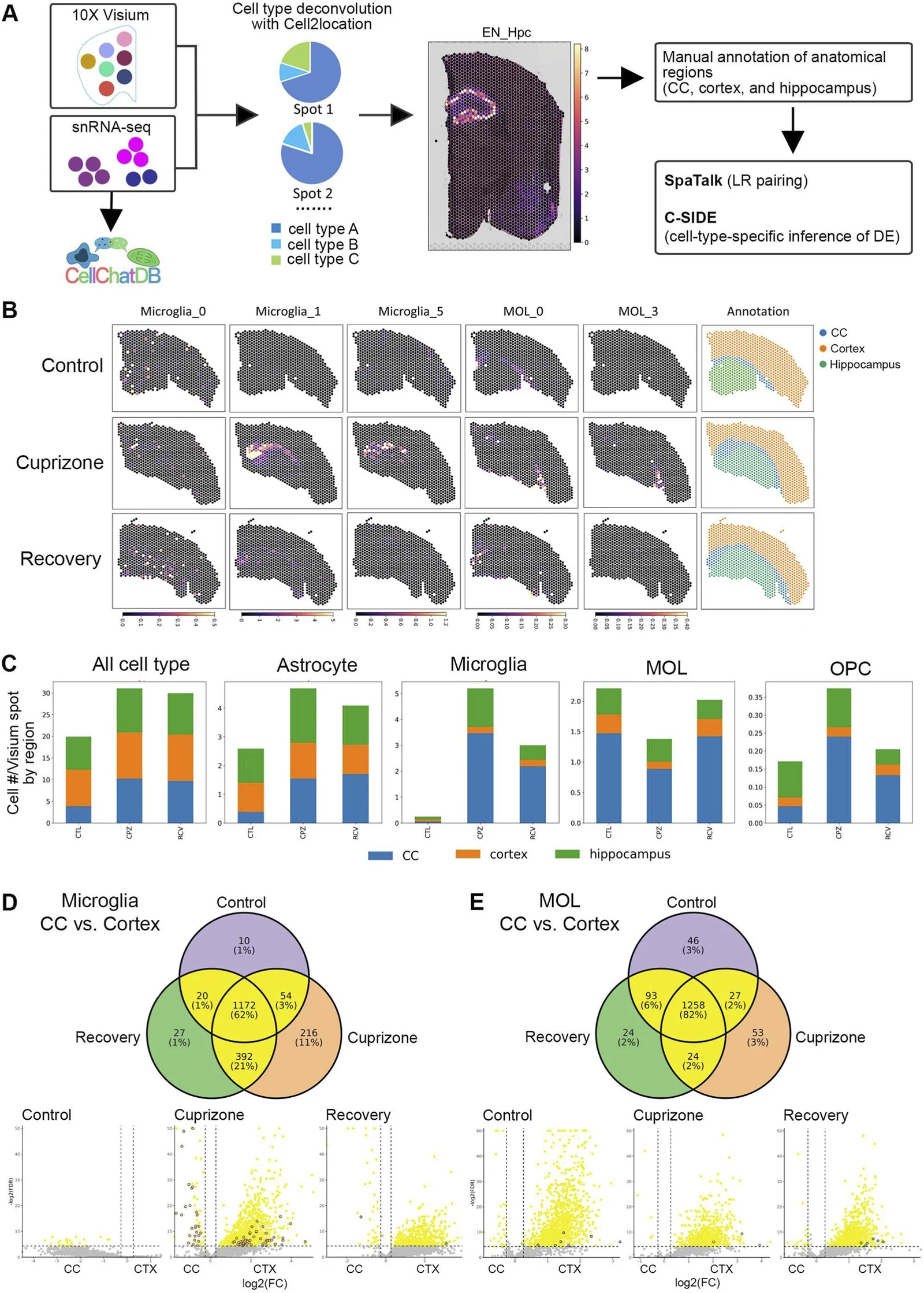
Deconvoluted ST data mapped CPZ-associated cell subclusters and demonstrated cell type abundance and expression heterogeneity across brain regions. **(A)** The workflow illustrating the steps involved in the ST data analysis. **(B)** Spatial plot showing the abundance of CPZ-associated subclusters of microglia and oligodendrocytes using cell2location. **(C)** Stacked bar plot representing the abundance of different cell types across various treatments in different brain regions. **(D)** Differential expression of microglial genes in CC compared to the cortex across different treatments represented by genes detected in each condition in the Venn diagram (upper) and DEGS volcano plots (lower), with yellow genes being DEGs shared in at least 2 conditions and purple, orange, and green points representing DEGs unique to CTL, CPZ, and RCV respectively, and **(E)** Differential expression of MOL genes in the CC compared to the cortex across different treatments represented by Venn diagram (upper) and volcano plots (lower) as described in **(D)**.

Since the 10X Visium Spatial Gene Expression platform captures multiple cells per spot, we used the snRNA-seq data as a reference to deconvolute cell types at each spot by cell2location (Figure 5A) (Kleshchevnikov et al., 2022). As expected, EN-Hpc neurons were mapped to the hippocampal region after deconvolution (Figure 5A). To take advantage of ST to spatially resolve gene expression in each cell type, we manually annotated regions of interest (the CC, cortex, and hippocampus) on each Visium section, using adjacent histology section images as references (Figure 5A).

CPZ-associated microglial subclusters 1 and 5 were mapped mostly to the medial CC on the representative CPZ section, and these subclusters were almost nonexistent on the CTL or RCV sections (Figure 5B; Supplementary Figure S6). This finding is consistent with the IHC data showing that microglial activation is most prominent in the CC under CPZ (Supplementary Figure S1). In contrast, homeostatic microglia subcluster 0 did not concentrate in any brain region. CPZ-associated oligodendroglia subcluster 3 was mapped mostly onto the lateral CC on the representative CPZ section and was almost nonexistent on the CTL or RCV sections. Oligodendroglia subcluster 0 was mapped to the entire CC region in CTL and to the medial CC region in the RCV section. Interestingly, MFOLs from the oligodendrocyte lineage mostly mapped to the RCV section (Supplementary Figure S4A), suggesting an active remyelination process.

We next investigated the abundance of each cell type by region under each condition. In general, there was a substantial increase in the number of cells per Visium spot in the CC (Figure 5C) compared with other brain regions. Microglia exhibited a sharp increase in cell abundance in the CC and hippocampus in the CPZ group. The abundance of microglia remained high in the RCV group, suggesting incomplete reversal of the phenotype to that of the CTL group. On the other hand, the MOL abundance in the CC, cortex, and hippocampus was reduced in CPZ but recovered to the CTL level in RCV, suggesting a robust remyelination process after CPZ withdrawal. The OPC number in the CC continued to remain high under the RCV condition.

Finally, we investigated region-specific cellular responses to CPZ. Microglial populations in the cortex and CC had their own gene signatures under control conditions. Under the CPZ condition, both cortical and CC microglia responded by increasing gene expression. In the microglia CC vs. cortex contrast, 11% of genes were unique to the CPZ condition (Figure 5D). MOLs located in the CC and cortex also had different gene signatures in the CTL condition, and both responded mildly to CPZ (Figure 5E). Overall, we found region-specific and cell type-specific responses under the CPZ condition, which is further supported by astrocytes (Supplementary Figure S4B) and OPC populations. OPCs located in the CC and cortex showed similar gene expression signatures, and they did not seem to respond to CPZ by changing their expression even though the number of OPCs markedly increased under CPZ conditions (Figure 5C; Supplementary Figure S4C).

### SpaTalk predicts heterogeneous cell‒cell communication in the cortex and CC

We used SpaTalk to infer spatially resolved cell‒cell interactions from the deconvoluted Visium data (Shao et al., 2022) and focused on interactions among 4 cell types: microglia, astrocytes, MOL, and OPC. In the cortex, the interaction between astrocytes and microglia in the CPZ had the greatest number of identified receptor pathways, including death receptor, TNF, and interleukin family signaling pathways. The interaction between microglia and MOL in the CPZ condition was associated with the second highest number of receptor pathways identified, including interleukin family signaling, ERK1/2 activation, and the Wnt signaling pathway. Most receptor pathways in the cortex were identified in the CPZ group, while the MOL-to-OPC and OPC-to-MOL-enriched receptor pathways were identified in the CTL group (Supplementary Table S1).

In the CC, fewer significant receptor pathways were identified than in the cortex, and astrocyte-to-microglia interactions continued to be associated with the CPZ condition (Supplementary Table S2). Selected receptor pathways of interest from SpaTalk with a representative LR pair are plotted in Supplementary Figure S5. Finally, we applied the same filters to CellChat and SpaTalk and identified 20 consensus LR pairs in the CC under the CPZ condition (Table 1). Growth factor and phagocytic pathways were enriched in 11 out of the 20 consensus LR pairs, and microglia were the major sender cell type.

**TABLE 1:** Common LR pairs in CC under the CPZ condition predicted by both CellChat and SpaTalk.

Sender	Receiver	Ligand	Receptor
Astrocyte	Microglia	Fgf1	Fgfr2
Astrocyte	Microglia	Gas6	Axl
Astrocyte	Microglia	Pros1	Axl
Astrocyte	Microglia	Vegfa	Flt1
Astrocyte	Microglia	Vegfb	Flt1
Microglia	Astrocyte	Fgf2	Fgfr1
Microglia	Astrocyte	Fgf2	Fgfr2
Microglia	Astrocyte	Fgf2	Fgfr3
Microglia	Astrocyte	Gas6	Axl
Microglia	Astrocyte	Igf1	Igf1r
Microglia	Astrocyte	Pdgfa	Pdgfrb
Microglia	mol	Fgf2	Fgfr2
Microglia	mol	Igf1	Igf1r
Microglia	opc	Fgf2	Fgrf2
Microglia	opc	Igf1	Igf1r
Microglia	opc	Pdgfa	Pdgfra
mol	Microglia	Gdf15	Tgfbr2
mol	Microglia	Vegfa	Flt1

### The CPZ model exhibits moderate translatability to MS chronic active lesions

**FIGURE 6:**
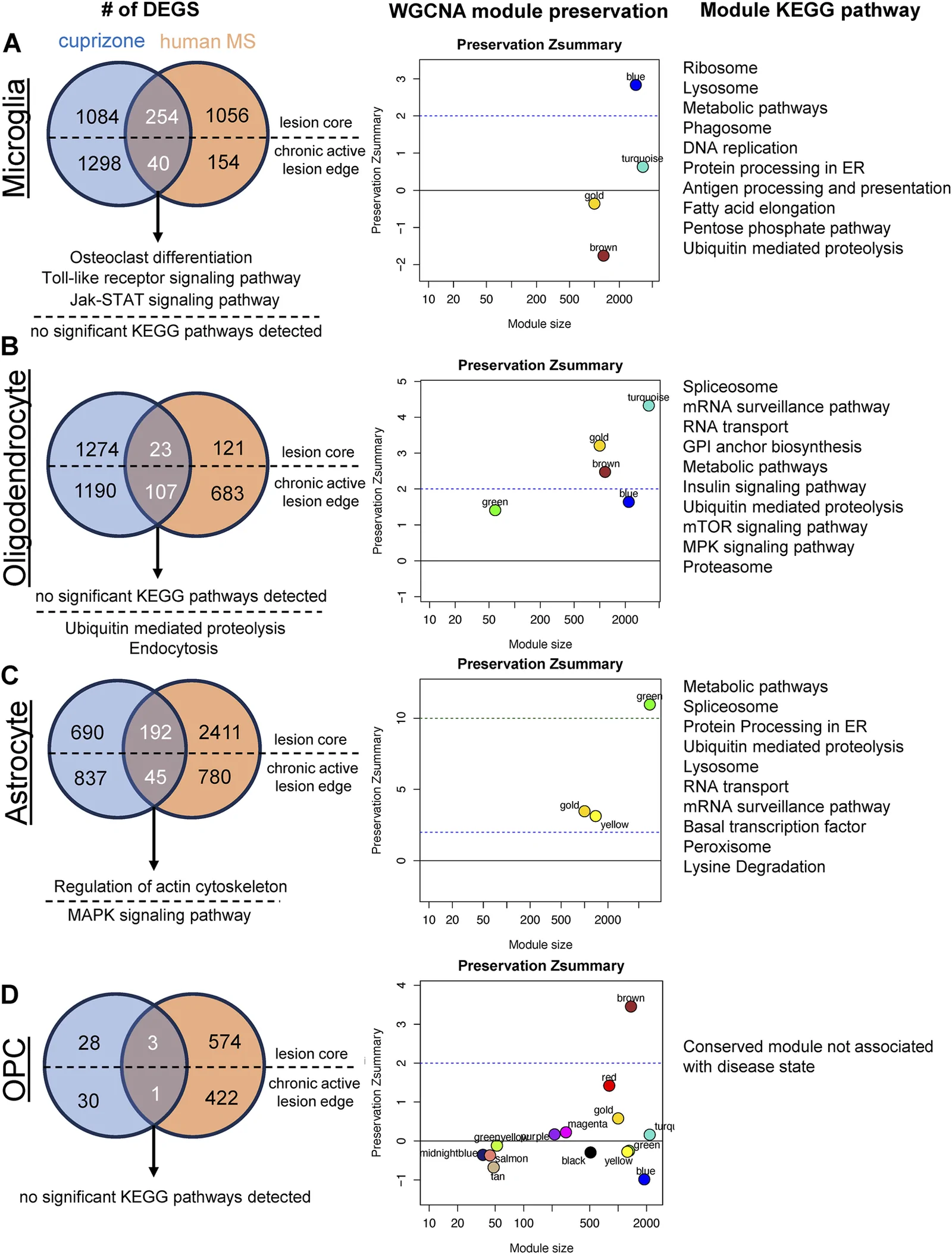
Translatability analysis revealed different levels of conservation of disease-associated modules driven by cell types between CPZ and human MS. **(A)** Comparison of DEG lists from CPZ (blue) and human MS (orange) in microglia (left). The number of genes shared between the two datasets is represented as white numbers at the intersection of the Venn diagram. DEGs from the CPZ were compared to those from two different lesion locations in the human data, either “control vs. lesion core” (above dotted line) or “control vs. chronic active lesion edge” (below dotted line). KEGG pathway results using the shared genes as input are shown below each Venn diagram, with a dotted line again separating human comparisons of “lesion core” or “chronic active lesion edge.” hdWGCNA results showing the number of genes and preservation score of modules from microglia calculated from the CPZ data. A module preservation score >2 (dotted line) is considered moderately conserved between mice and humans, while a score >10 is considered highly preserved. KEGGprofile results of the top preserved disease-associated module for microglia (right). **(B)** Comparison analysis of the MOL. **(C)** Comparison analysis of astrocytes. **(D)** Comparison analysis of OPCs. The moderately preserved brown module in the OPC cell type was not associated with disease and thus was not analyzed further.

Lastly, we explored the translatability of the CPZ model by performing an integrated analysis of our dataset with a published MS RNA-seq dataset that profiled chronic active lesions in humans (Absinta et al., 2021). MS white matter lesions can be classified into various types based on the infiltrating immune cells, the activity of monocyte-derived macrophages and resident microglia, and the degree of demyelination (Kuhlmann et al., 2017). Among white matter lesions, disease disability correlates with chronic active lesions that have iron-laden microglia present at the lesion rim, which can be visualized by specific magnetic resonance imaging (MRI) sequences in the clinic (Kaunzner et al., 2019; Absinta et al., 2021). We reanalyzed both human and mouse datasets using the same methods presented in this study and calculated DEGs between the disease and control groups to determine the level of DEG conservation between the two datasets. To take advantage of the lesion segmentation defined by Absinta *et al.*, we calculated DEGs of both “lesion core vs. control” and “chronic active lesion edge vs. control” for each glial cell type (Figure 6, left). We then calculated the overlap in DEGs between mice and humans and subsequently performed KEGG analysis on DEGs common to both species. We found pathways that were conserved between the CPZ mouse and human MS samples depending on both the cell type and the lesion architecture investigated. For example, gene expression changes in microglia in the CPZ condition were more in concordance with gene expression changes in the MS lesion core, whereas changes in oligodendrocyte in CPZ were more in concordance with changes at the lesion edge.

We also used an alternative approach to investigate the translatability by using a variation of weighted correlation network analysis designed for single-cell data (hdWGCNA) (Morabito et al., 2021; Morabito et al., 2023). We used hdWGCNA to define disease-associated modules that were both correlated with the disease state and preserved between the two species (Langfelder et al., 2011). We found disease-associated modules in the CPZ model that showed moderate to strong preservation in humans depending on the cell type investigated (Figure 6, middle column). Astrocytes had the highest preservation scores of disease-associated modules, while MOL, OPC, and microglia showed moderate to low preservation between the CPZ and the Absinta *et al.* datasets. These disease-associated and preserved modules showed KEGG pathway enrichment that was shared among cell types, such as metabolic pathways (in astrocytes, microglia, and the MOL), protein processing in the endoplasmic reticulum (in astrocytes and microglia), and ubiquitin-mediated proteolysis (in astrocytes, microglia, and the MOL) (Figure 6, right column). In the future, this workflow can be applied to compare different MS lesion types with the CPZ model or others to understand the validity of mouse models.

## Discussion

In this study, we profiled the CPZ model by “space” and “time” using ST along with an array of bioanalytical tools. We found that demyelination and neuroinflammation in the CPZ model were more prominent and widespread across the dorsal to ventral axis than previously identified by IHC. We identified oligodendroglia and microglia as two major cell types associated with de- and remyelination in the model and we mapped CPZ-associated subclusters of oligodendroglia and microglia in the CC, which shows stereotypical demyelination. In the RCV group, while MOL almost normalized to the control state, microglia continued to be associated with the CPZ phenotype, i.e., demyelination. We inferred cell‒cell interactions using both snRNA-seq and ST data and found that the phagocytosis, growth factor, and chemokine pathways were enriched under the CPZ condition. ST is a fast-emerging field that enables high-throughput investigations of spatially resolved transcripts (Liu et al., 2021; Rao et al., 2021). Prior to the development of ST technology, region-specific gene expression studies in MS brains relied heavily on manual dissections (Hendrickx et al., 2017; Jäkel et al., 2019; Absinta et al., 2021). Manual dissection of lesions is sufficient to identify major biological heterogeneity but lacks fine resolutions. Based on our study of the CPZ model, ST appears to be an appropriate tool for investigating detailed cellular and molecular substrates underlying MS lesion evolution as well as predicting cellular interactions.

Our findings identified oligodendroglia and microglia as cell types of interest during de- and remyelination. This result is consistent with the IHC results showing that demyelination (oligodendrocytes) and gliosis (microglia) are two prominent features of the CPZ model, particularly in CC lesions (Praet et al., 2014). However, considering proposed functions of astrocytes in the experimental autoimmune encephalomyelitis (EAE) model and MS (Ponath et al., 2018; Wheeler et al., 2020; Wheeler et al., 2023), it is unexpected that astrocytes were not significantly associated with any experimental conditions, and no disease-associated subclusters were identified in our dataset. It is possible that (1) the recovery time point (2 weeks after CPZ withdrawal) is too early to capture a full astrocytic response in our CPZ model, or (2) astrocyte involvement in demyelination pathology is animal model dependent, as the EAE model is more inflammatory in nature. (3) It has been suggested that reactive astrocytes are highly heterogeneous and the responses are context/region dependent. Moreover, there is co-existence of many pathology-related reactive sub-states of astrocytes (Patani et al., 2023). Thus, it is possible that the astrocyte population in the CPZ model undergoes a widespread and substantial gene expression change that is moderately consistent with the changes in MS without forming identifiable CPZ-associated subclusters. Nonetheless, CellChat and SpaTalk predicted numerous potential LR pairs between astrocytes and microglia, highlighting the interactions between the 2 cell types and the neuroinflammatory pathways associated with these interactions. Interestingly, astrocytes had the highest preservation scores of disease-associated modules with MS among all the cell types analyzed by hdWGCNA for model translatability. Although we did not identify astrocytes as “pathogenic” cells in the CPZ model, this finding suggests that astrocyte transcriptomic changes in the model are still in line with the changes in MS.

The growth factor and phagocytic pathways were predicted by both CellChat and SpaTalk as potential LR interactions in the CC under the CPZ condition. The role of growth factors in MS has long been recognized (Webster, 1997), and approximately three-quarters of the LR pairs in our study are related to growth factor signaling. While experimental validation of the identified LR interactions is out of scope in this study, we propose growth factor pathways as a high priority direction for future functional investigations, particularly the VEGF and FGFR pathways. In addition to blood vessel remodeling, the VEGF pathway is involved in pathogenic glial responses (Rothhammer et al., 2018) and meningeal lymphatic dysfunction (das Neves et al., 2024) that are relevant to MS. Based on our findings, further investigation of the contributions of VEGFA-FLT1 and VEGFB-FLT1 cross-talks across space and time will help elucidate the disease mechanisms at the CNS borders. Through FGFR signaling, FGFs are involved in various biological and pathological processes, and FGF1 and FGF2 are the main mitogens in the CNS (Beenken and Mohammadi, 2009). Evidence has shown that FGF1 and FGF2 are expressed in MS lesions with a remyelination phenotype (Clemente et al., 2011; Mohan et al., 2014), and it has also been shown that FGF signaling is critical for regulating oligodendrocyte and myelin regeneration (Furusho et al., 2015). Thus, recombinant FGFs have been proposed to be a potential therapeutic approach for MS. On the other hand, oligodendrocyte-specific deletion of FGFR1 and FGFR2 results in a protective effect in the EAE model; therefore, FGFR inhibitors have also been proposed for MS (Rajendran et al., 2023). Disentangling the complex biology of FGF signaling will be critical for MS therapy development and utilizing an ST approach can provide much needed information in terms of cell-cell interaction *in situ*.

The remaining LR pairs are related to phagocytic pathway signaling. Myelin debris have been shown to inhibit remyelination (Kotter et al., 2006), and it has also been suggested that a dysfunctional myelin sheath under inflammatory attack could pose a greater risk of axonal degeneration (Schaffner et al., 2023). Therefore, a precise and efficient phagocytic program to clean myelin debris is critical for successful remyelination. In nondemented individuals from the Swedish BioFINDER-2 cohort, increased levels of microglial markers in the CSF, such as soluble TREM2, AXL, MERTK, GAS6, LPL, CST7, SPP1 and CSF1, are associated with slower cognitive decline (Pereira et al., 2022). In our dataset, Gas6-Axl and Pros1-Axl were in the consensus LR pair list from CellChat and SpaTalk, while Gas6-Mertk and ApoE-Trem2 were predicted from the CellChat analysis. Gas6 and Pros1 are ligands for the TAM (Tyro3, Axl, and Mertk) receptor tyrosine kinase family, and their signaling regulates inflammation, cell growth, and clearance of debris (Bellan et al., 2016). The role of phagocytosis-related proteins in MS and whether they can be potential therapeutic targets are less clear than those in Alzheimer’s disease research (Weinger et al., 2009). However, studies in the CPZ model have suggested that both Mertk and Trem2 play roles in myelin debris clearance and subsequently promote remyelination (Cignarella et al., 2020; Shen et al., 2021). A detailed profiling of phagocytic proteins in MS cerebrospinal fluid to see how they correlate with MS disease severity and progression could be a worthwhile effort. Overall, the LR pairs prediction in our study showed similarities to cell‒cell interactions identified from human MS cortical ST data (Kaufmann et al., 2022). For example, growth factor pathway interactions, such as PSAP-GPR37L1, CSF1-CSF1R, and PDGFC-PDGFRA, which represent approximately one-third of the pathways identified in the MS cortical ST data, were also present in our CPZ dataset. One-fourth of the pathways in the MS cortical ST data were grouped as anti-inflammatory, such as the PROS1-AXL interaction, which was also predicted in our dataset. These findings further support the moderate translatability of CPZ model to human MS.

The advantage of our study is that we employed a hypothesis-free whole-genome approach, but we also encountered some study limitations. First, while cell‒cell interaction investigations in our study provided novel findings, the LR pairs did not immediately predict therapeutic directionalities. Due to the scope of the study, functional validation will be performed in a separate investigation. Second, it is important to acknowledge that 10X Visium technology lacks single-cell resolution, which is a key limitation. To address this issue, we used cell2location, which integrates snRNA-seq data with spatial data to deconvolute cell types, providing insights into the abundances of different cell types within the 10X Visium spots. Although this technique does not achieve true single-cell resolution, it represents a crucial step toward a more detailed characterization of the cellular landscape. Third, the sample size in our study is small. Some potential results may not reach statistical significance due to the small sample size. Conversely, significant findings from our study should also be validated in a larger study or using orthogonal methods. Finally, while we believe that the CPZ model captures some of the salient demyelination features of MS, we cannot exclude direct effects of CPZ on cells other than the oligodendrocyte population or effects from CPZ on biological processes unrelated to demyelination, which could introduce noise into the analyses (Skripuletz et al., 2011).

## Materials and methods

### Cuprizone model

All procedures involving animals were reviewed and approved by the Biogen Institutional Animal Care and Use Committee and conducted under protocol 801 in accordance with relevant guidelines and regulations. All methods are reported in accordance with ARRIVE guidelines (https://arriveguidelines.org). Male wild-type C57BL/6J mice aged about 10 weeks were purchased from Charles River Laboratories (stock number 027C57BL/6). The research animals were housed under specific-pathogen-free (SPF) conditions in an AAALAC accredited facility according to Biogen’s institutional animal care and use committee (IACUC) protocol with a 12 h–12 h light–dark cycle and environmental conditions controlled at 72°F/22 °C and 40%–60% humidity. To establish the CPZ model, customized 0.3% (w/w) cuprizone chow was made by mixing cuprizone powder (Sigma‒Aldrich 14690) with control base chow (AIN-76A) at Research Diets Inc. All food chow (CPZ and control) was stored in vacuum bags at 4 °C, and fresh food chow was replaced weekly during the study. In the CPZ group, the animals were fed cuprizone chow for 4 weeks. For the recovery group, animals were fed cuprizone chow for 4 weeks, followed by 2 weeks of control base chow for recovery. For the control group, animals were provided with control base chow for 6 weeks. The body weight of each mouse was closely monitored weekly throughout the study, and mice with ≥30% weight loss were excluded.

### Tissue collection for sequencing studies

For snRNA-seq, spatial transcriptional sequencing, and bulk RNA-seq studies, the CPZ-treated mice were sacrificed, and brain tissues were collected at either week 4 (n = 3, CPZ group) or week 6 (n = 3, recovery group). The control mouse brain tissues were collected at week 6 (n = 4, control group). At each time point, the mice were euthanized by CO_2_ inhalation, followed by transcardiac PBS perfusion and brain tissue dissection. Each brain was cut sagittally to separate the left and right hemispheres; one brain hemisphere was trimmed and embedded in OCT for snRNA-seq or spatial transcriptomic study, and the other hemisphere was snap frozen for bulk RNA-seq analysis (Figure 1). To enrich for cells from the corpus collosum and cortex, only the top half of the coronal section, which included the dorsal cortex, CC, and hippocampus, was collected for snRNA-seq.

### Immunohistochemistry (IHC)

A separate cohort of animals was used for the IHC study. Brain tissues were dissected and fixed for 24 h in 10% NBF at 4 °C and then transferred to ice-cold PBS for coronal sectioning on a vibratome. Coronal brain slices of 40 µm thickness from bregma −1 mm to −2.5 mm were collected and stored in 30% sucrose/PBS buffer at −20 °C. For staining, 3 slices spanning 400 µm apart were selected from each animal. For staining, brain slices were thawed in PBS at room temperature and then blocked with blocking solution (0.5% Triton, 5% goat serum, and 0.1% BSA in PBS) for 1 h at room temperature. Primary antibodies were diluted in blocking solution and incubated with the slices overnight at 4 °C. The corresponding secondary antibodies were applied for 1–2 h at room temperature. After incubation with secondary antibodies, the slices were washed and mounted onto glass coverslips with Prolonged Gold Mounting Solution (Thermo Fisher, P36931). The following primary antibodies were used: mouse anti-MBP (BioLegend, 88,408, 1:500), rat anti-CD68 (Bio-Rad, MCA1957, 1:300), and chicken anti-GFAP (Thermo Fisher, PA1-10004, 1:500). The following secondary antibodies were used: goat anti-chicken IgY 647 (Thermo Fisher, A32933, 1:500) and donkey anti-rat IgG488 (Thermo Fisher, A21208, 1:500).

Images were obtained using a Zeiss LSM 710 confocal microscope with a ×20 objective and Z-stack of 10 planes at 1 µm intervals. Individual fields of each slice were tiled into a large image.

### Tissue sectioning and preparation for spatial transcriptomics

Frozen sections for ST profiling were collected using a Leica CM1520 cryostat with a Leica Surgipath high profile blade at −23 °C. Ten-micron-thick sections were collected from mouse brain hemispheres embedded in OCT (TissueTek) on Visium Spatial Gene Expression slides (10X Genomics Slide Kit, Ref No. 1000185). Tissue sections were carefully collected to allow accurate positioning within individual capture areas of Visium slides following a randomized collection scheme across multiple Visium slides to help control for (1) slide-to-slide batch effects by ensuring that a section from each treatment group was present on each slide and (2) the potential impact section location on the slide by ensuring that sections from a given treatment group were not in the same position or order on every slide. The frozen slides were processed for spatial transcriptomic sequencing according to the manufacturer’s instructions. Briefly, the sections were heated at 37 °C for 1 min and then fixed in methanol at −20 °C for 30 min. Following fixation, the samples were stained with hematoxylin and eosin. Brightfield images were collected on an Olympus VS120 Virtual Slide Microscope equipped with a VC50 camera using a UPLSAPO ×20 objective. After imaging, tissue permeabilization was carried out using the supplied permeabilization buffer at 37 °C for 6 min. Permeabilization time was selected based on a prior optimization experiment in mouse brain tissues using Visium Spatial Tissue Optimization slides (10X Genomics, Ref. No. 1000193). Following permeabilization, reverse transcription and second strand synthesis reactions were carried out on slides before elution of the resulting cDNA into separate sample PCR tubes according to Visium guidelines. qPCR was performed on 1 µL of eluted cDNA as described in the Visium Spatial Gene Expression Reagent Kits User Guide (Revision-B) and was run on an Applied Biosystems QuantStudio 12K Flex qPCR instrument (Applied Biosystems™ 4471087). Individual sample cDNA PCR amplification cycles were selected and run based on sample cDNA Ct. Total yields of amplified cDNA ranged from 300 to 550 ng. Sequencing library generation was performed using the 10X Genomics Library Construction Kit (PN-1000190) following the manufacturer’s instructions with 13 cycles of PCR based on the cDNA input used. Library quality was measured using a PerkinElmer LabChip GXII with a Hi Sens Lab Chip (Perkin Elmer 760517) and DNA High Sensitivity Reagents (Perkin Elmer CLS760672), which showed an average spatial gene expression library size of 473 bp. Libraries were normalized and pooled before loading at 300 pM on an Illumina NovaSeq 6000 S2 flow cell with paired-end sequencing parameters of Read1-28 bp × i7-10 bp × i5-10 bp × Read2-120 bp.

### Bulk RNA sequencing and data processing

Bulk RNA-seq libraries were prepared using the Kapa mRNA HyperPrep Kit (Kapa Biosystems KK8581) according to the manufacturer’s instructions. Briefly, 100 ng of RNA was fragmented for 6 min at 94 °C, followed by RT, second strand synthesis, A-tailing, adaptor ligation, and amplification with 14 cycles of PCR. Libraries were quantified on a LabChip GXII as described above. Libraries were normalized, pooled, and sequenced on an Illumina HiSeq2500 platform, with an average depth of 9 million 50 bp paired-end reads. Run quality metrics were assessed using Illumina’s BaseSpace run summary tool. Bulk RNA analysis was performed using the RNASequest pipeline (Zhu et al., 2023) following the tutorial provided in the manuscript.

### Bulk RNA-seq integration with scRNA-seq

We applied Scissor (Sun et al., 2022) (https://github.com/sunduanchen/Scissor) to integrate the bulk and snRNA-Seq datasets. It utilizes the phenotypes (CPZ, RCV, and control) collected from bulk assays to identify the most highly phenotype-associated cell subpopulations from single-cell data.

### Nuclei isolation

Nuclei isolation was performed as described previously (Hrvatin et al., 2019). Tissue was dounced in 0.5 mL Hyman Lysis Buffer (0.32 M Sucrose, 5 mM CaCl_2_, 3.0 mM MgAc_2_, 0.1 mM EDTA, 10 mM Tris-HCl, pH 8.0, 1 mM DTT, 0.1% Triton X-100, 1/1000th volume Promega RNasin Plus RNase Inhibitor) 10X with loose pestle, followed by 10X with tight pestle. Following douncing, 1 mL of Hyman Low Sucrose Buffer (0.32 M Sucrose, 5 mM CaCl_2_, 3.0 mM MgAc_2_, 0.1 mM EDTA, 10 mM Tris-HCl, pH 8.0, 1 mM DTT, 1/1000th volume Promega RNasin Plus RNase Inhibitor) was added to the homogenate, and passed through a 70 μm filter, followed by a 1 mL Hyman Low Sucrose Buffer rinse of the dounce and filter. Homogenates were spun at 600 × g for 5–10 min at 4 °C. After supernatant aspiration, pellets were gently resuspended in 5 mL Complete Buffer HB (0.25 M sucrose, 25 mM KCl, 5 mM MgCl_2_, 20 mM Tricine-KOH, pH 7.8, 1 mM DTT, 0.15 mM spermine, 0.5 mM spermidine, 1/500th volume Promega RNasin Plus RNase Inhibitor), followed by addition of 5 mL Working Solution (50% Iodixanol: 5 volumes OptiPrep Density Gradient Medium, 1 volume Diluent Buffer (150 mM KCl, 30 mM MgCl_2_, 120 mM Tricine-KOH, pH 7.8), 1/1,000th volume Promega RNasin Plus RNase Inhibitor). In a 12 mL Corex tube, 1 mL 40% iodixanol (Working Solution diluted in Complete Buffer HB, 0.042% BSA), and 30% iodixanol (Working Solution diluted in Complete Buffer HB, 0.021% BSA) were layered, followed by 10 mL of resuspended sample. The samples were spun in an ultracentrifuge at 10,000 × g for 18 min at 4 °C with the brake off. After spinning, the fatty myelin top layers were vacuum aspirated, followed by a 25% iodixanol layer and approximately 1/3 of the 30% iodixanol layer. Approximately 0.5 mL of nuclear sample band at the 30%–40% iodixanol interface was collected, transferred to an Eppendorf tube and mixed by pipetting. Nuclei were stained with DAPI and counted with a Countess II FL. Nuclei suspensions were diluted to 1000 nuclei/μL in RB (PBS + 2% BSA + 1/1000th volume Promega RNasin Plus RNase Inhibitor).

### snRNA sequencing

Single-nuclei RNA-seq was performed using the 10X Genomics Chromium Next GEM Single Cell 3′ Kit v3.1 (PN1000268), Chromium Next GEM Chip G (PN1000127), and Dual Index Kit TT Set A (PN1000215). Nuclei suspensions were loaded on the 10X Chromium instrument for recovery of 5000 nuclei per sample. cDNA synthesis, PCR, and library preparation were performed according to the manufacturer’s instructions, with 12 cycles of PCR for cDNA amplification, and 15 cycles for library amplification. cDNA and libraries were quantified on a LabChip GXII as described above. Libraries were normalized, pooled, and loaded at 300pM on an Illumina NovaSeq 6000 S4 flowcell with paired end sequencing parameters of Read1-28bp × i7-10bp × i5-10bp × Read2-90bp targeting an average depth of 85,000 reads per cell.

### snRNA-seq data preprocessing and quality control

FASTQ files were generated from Illumina bcl files using CellRanger mkfastq. CellRanger count was used to identify UMI and cell barcodes and to align reads to the mouse genome assembly mm10, with intronic counts included. To remove ambient RNA from the gene expression matrices, the ‘remove-background’ function of CellBender was used (Fleming et al., 2023; Janssen et al., 2023). In addition, CellBender identifies and removes empty droplets. The CellRanger output file ‘raw_feature_bc_matrix.h5’ was used as the input file for CellBender. The ‘Estimated Number of Cells’ from CellRanger was used as the ‘expected-cells’ parameter, while a value in the plateau of the barcode rank plot was used as the ‘total-droplets-included’ parameter. Potential doublets were identified and removed using scDblFinder (Germain et al., 2021). Filtered barcode matrices were concatenated using Scanpy (Wolf et al., 2018). QC metrics, including the number of detected genes, total counts, and percentage of counts from mitochondrial genes, were calculated for each barcode. Cell barcodes with fewer than 1000 detected genes, greater than 100,000 counts, or greater than 10% mitochondrial UMIs were filtered out from the dataset.

As an additional QC metric, intronic mapping rates were derived on a per-barcode basis using the BAM alignment files output by CellRanger count. Compared with cellular transcriptomes, nuclear transcriptomes have been demonstrated to show a greater proportion of reads mapping to intronic regions (Bakken et al., 2018), enabling the detection of nonnuclear cell barcodes representing contaminating cytoplasmic transcripts.

### snRNAseq cell type annotation and subclustering

Cell type labels were assigned using label transfer from E-MTAB-11115 (Kleshchevnikov et al., 2022), with region-specific astrocyte subtypes merged into a single astrocyte label. Oligodendrocyte-lineage cell types were further clarified by label transfer from GSE75330 (Marques et al., 2016) after merging major cell types from this reference. Filtered UMI counts for E-MTAB-11115 were downloaded from ArrayExpress along with cell type annotations. The UMI was then processed by Seurat (Hao et al., 2021), LIGER (Welch et al., 2019) and Harmony (Korsunsky et al., 2019) integration on different sample libraries. The kBET (Büttner et al., 2019) evaluation revealed that LIGER achieved the best performance. Thus, the Seurat SCT and LIGER layout (PCA) was used to generate the Seurat azimuth reference. In parallel, the cells were sequentially subclustered *via* Harmony and Louvain clustering.

To investigate the heterogeneity within the microglial and oligodendrocyte populations, we subclustered these cell types using the Leiden clustering algorithm implemented in Scanpy. By applying a resolution parameter of 0.5, we identified 12 subclusters each for microglia and oligodendrocytes. Differential gene expression analysis was performed to identify marker genes that exhibited significant differences in expression between subclusters with the Wilcoxon rank-sum test. To visualize marker gene expression, we generated dot plots that depict the percentage of cells expressing the gene through dot size, and the color intensity represents the relative expression level.

### snRNA-seq DE analyses

snRNA-seq data were analyzed per cell type using the NEBULA-HL single cell DE method (He et al., 2021). For each cell type, 3 contrasts were considered: CPZ vs. Control, CPZ vs. Recovery, and Recovery vs. Control. Cell types with at least 10 cells in each of the biological replicates within the contrast were analyzed. The genes tested in each contrast group were expressed in at least 10% of the cells in both contrast groups. Mouse ID was used as a random effect in the model. Library size was used as an offset covariate.

### Pseudotime trajectory analysis and velocity visualization

We used scFates (Faure et al., 2023) for trajectory analysis. scFates combines a tree inference approach similar to SimplePPT (Mao et al., 2015) and a principal graph learning algorithm named ElPiGraph (Albergante et al., 2020) to calculate trajectories.

### 10x Visium spatial sequencing data analysis

The 10x Visium spatial sequencing data were processed using Space Ranger v1.1.0 (10x Genomics). The sequencing results (BCL files) were converted to fastq files with the Space Ranger mkfastq pipeline. The fastq files were then mapped to the mouse reference genome (GRCm38.p3) using the Space Ranger count pipeline, which produces a spot-barcoded expression matrix.

Next, we used SpaGCN (Hu et al., 2021), a graph convolutional network-based method, to identify spatial clusters based on both gene expression profiles and spatial locations. All the slides were aligned and normalized using the PASTE algorithm (Zeira et al., 2022). The MS gene signature selected for this study was based on consensus genes in multiple sclerosis signaling, myelination signaling and neuroinflammation signaling pathways from QIAGEN Ingenuity Pathway Analysis (IPA) that were identified in Figure 1E, including: *B2m, C1qa, C1qb, C1qc, C3, C3ar1, C4b, Ccl2, Cd86, Creb3l1, Cxcl10, Egr2, Eif4ebp1, Fos, Hck, H2-Q4, H2-Dmb1, H2-Oa, H2-Aa, H2-Ab1, H2-Eb1, Hmox1, Icam1, Idi1, Igf1, Il33, Irf7, Itgax, Itgb2, Itgb4, Itgb5, Mag, Mapk4, Mbp, Mobp, Mog, Mthfd2, Ncf2, Opalin, Parp14, Parp9, Plp1, Pycard, Rac2, Rnf213, Serping1, Slc6a12, Tgfbr1, Tlr2, Tlr3, Tlr7, Trem2, Tyrobp*.

Then, we used Cell2location (Kleshchevnikov et al., 2022) to spatially map cell types in the Visium spatial transcriptomics data. This package integrates single-cell RNA-seq (scRNA-seq) or single-nucleus RNA-seq (snRNA-seq) with spatial transcriptomics data to deconvolute cell types in each spot of the Visium slide. A reference mouse brain cell type signature representing gene expression profiles for each annotated cell type was estimated from snRNA-seq data using a negative binomial regression model. The parameters used for the model were batch size = 2500, train_size = 1, learning rate = 0.002, and max_epochs = 250. Then, the reference cell type signature was integrated with the processed 10X Visium data to deconvolute the absolute spatial abundance of each cell type on the spatial transcriptomics slide. The deconvolution model requires the user to input two hyperparameters: 1) the expected cell abundance (N_cells_per_location), which was set to 5, and 2) the regularization strength of the detection efficiency effect (detection_alpha), which was set to 200 (default). The model was trained for 30,000 iterations. The default settings were used for the remaining parameters.

### ST pseudobulk PCA

To perform pseudobulk PCA of the spatial transcriptomics data, spot counts for each gene were aggregated across all spots within each slice to form a gene-by-slice count matrix. After performing variance stabilizing transformation (vst), the top 500 most variable genes from the normalized matrix were used to generate PCs. The plot shown in Figure 1B was generated from data processed with DESeq2 (Love et al., 2014) after correcting for batch effects using ComBat-seq (Zhang et al., 2020).

### ST DE and pathway analysis

ST spot-level data were analyzed per cell type using the NEBULA-HL single-cell DE method (He et al., 2021). In this analysis, each spot was handled independently (spot-level information regarding spatial adjacency was not considered). Regional subsets of mouse brain regions, including the CC, cortex, and hippocampus, were manually annotated. H&E histology section adjacent to each ST section were collected and processed as an overlay image to guide the anatomical annotation. Regional subsets of mouse brain regions, including the CC, cortex, and hippocampus, were manually annotated by examining the ST spot images with histology images to include spots in appropriate brain regions. Allen Brain Atlas mouse reference atlas (https://mouse.brain-map.org/) was referenced to confirm the annotation. For each region, 3 contrasts were considered for DE analysis, namely, CPZ vs. Control, CPZ vs. RCV, and RCV vs. Control. Spots with fewer than 100 counts or greater than 45% mitochondrial gene counts were filtered out from each slice before analysis. Mouse ID was used as a random effect in the model. Library size was used as an offset covariate. DEGs from each cluster were analyzed by QIAGEN Ingenuity Pathway Analysis (IPA) for pathway discovery using Core Analysis. To compare pathways across clusters, all 4 clusters were subjected to Comparison Analysis.

### Cell‒cell interaction analysis

We applied CellChat (Jin et al., 2021) to understand the cell-cell interactions from the snRNAseq dataset. For ligand-receptor (LR) analysis, the scRNA-seq count matrix and corresponding metadata were loaded into a Seurat object for use in CellChat. The count matrix was normalized, and cell type identities were assigned to cells. The mouse specific CellChat LR interaction database ‘Secreted Signaling’ was selected. CellChat with default settings was used to perform cell-cell interaction (CCI) analysis and infer the CCI network by computing cellular and signaling pathway communication probabilities (strength). The method truncatedMean was used to calculate the average gene expression per cell group. Each comparison (CPZ vs. Control, CPZ vs. Recovery, Recovery vs. Control) was analyzed separately and then merged into a single CellChat object. Differential LR combinations within the CCI network were identified in CellChat by comparison of the communication probabilities, and then those results were extracted for the cell type pairs of interest.

We applied SpaTalk (Shao et al., 2022) to infer cell‒cell interactions by enriching receptor pathways from the ST dataset. SpaTalk was run on three spatial sections per condition, with one section per biological replicate. Scaled cell type deconvolution weights precalculated by cell2location were used to deconvolve spots into cell types that were derived from the scRNA-seq reference data. Deconvoluted spots from two annotated regions, the cortex and CC, were analyzed in each section. Cell type assignments per spot were made using the default recommended size for 10X Visium 55-micron spots. Default SpaTalk spot-level analysis parameters were used to calculate cell‒cell LR interactions and to perform enriched LR receptor pathway analysis. Overlapping significant cell‒cell LR interaction pairs from both CellChat analysis based on snRNA-seq data and SpaTalk analysis based on ST data were used for downstream comparisons between CPZ and control conditions. The following set of 8 custom LR interaction pairs relevant to MS were added to the overlapping set: Il34-Csf1r, Apoe-Trem2, Pecam1-Pdgfra, Th-Drd1, Th-Drd2, Ddc-Drd1, Ddc-Drd2, and Spp1-Cd44.

### Translatability and hdWGCNA

For comparative DEG analyses between CPZ and the human dataset, we first reprocessed the data from Absinta et al. (2021) (see snRNA-seq data preprocessing and quality control) and called DEGs between control and MS tissues using Nebula (see snRNA-seq DE Analyses). For the human dataset, we performed DEG analysis on both the “control vs. lesion core” and control vs. “chronic active lesion edge” datasets to take advantage of the different lesion areas provided by Absinta *et al.* We identified genes that were upregulated in the disease state, with an FDR <0.05 for both datasets. We then converted mouse gene IDs into their human orthologs and counted the number of distinct and overlapping DEGs between the two datasets on a cell type basis. For a given cell type, DEGs that were common between CPZ and humans were used as input to the R package KEGGprofile for pathway analysis (https://github.com/slzhao/KEGGprofile/blob/master/DESCRIPTION).

Single-cell WGCNA was performed according to hdWGCNA (Morabito et al., 2021; Morabito et al., 2023) with modifications to regress out pseudobulk covariates, such as age and sex. The working principle of hdWGCNA is to first create pseudobulk samples of single-nucleus data based on kNN and subsequently identify groups of coexpressed genes (“modules”) for each cell type. Briefly, raw pseudobulk expression was calculated from specific cell populations by aggregating UMI counts from 50 cells using a k-nearest neighbor approach. We then normalized the raw count data by variance stabilizing transformation (VST) implemented by DESeq2. We regressed out confounding features such as total UMI count, mitochondrial percentage, and intronic rate. We then applied WGCNA to identify coexpressed genes (modules) using the normalized count data. Briefly, WGCNA starts by constructing a topological overlap (TO) matrix (Zhang and Horvath, 2005). To this end, we used weighted correlation with individual sample weights determined as described above and the “signed hybrid” network in which negatively correlated genes are considered unconnected. We used module eigengenes (MEs), the first principal component of the module’s gene expression matrix, to represent the overall expression patterns of the corresponding coexpression modules. The disease correlation score was defined as the Pearson correlation coefficient between the module eigengenes and disease status. Finally, we performed module preservation analysis using the modulePreservation function in WGCNA. The level of translatability between species is defined by the module preservation score (Langfelder et al., 2011). The output consists of “modules” of genes with similar trajectories, including scores to assess both correlation with disease (module correlation) and preservation between mice and humans (Zsummary). We considered modules with a disease correlation score >0.7 and a Zsummary >2 to be associated with disease and preserved between species. For pathway analysis, we used the module as an input for the KEGGProfiler.

## Data Availability

The datasets presented in this study can be found in online repositories. The names of the repository/repositories and accession number(s) can be found below: https://www.ncbi.nlm.nih.gov/geo/, GSE255371.
