# Supplementary material for: Spatial transcriptomics reveal heterogeneous cell‒cell interactions among brain regions in cuprizone model consistent with multiple sclerosis lesions
Source: Front Bioinform. 2026 Aug 6;6:1832826. doi: 10.3389/fbinf.2026.1832826 (PMC13490942; doi:10.3389/fbinf.2026.1832826)
Supplement: Supplementary file 1 [file DataSheet1.docx]

**Spatial transcriptomics reveals heterogeneous cell‒cell interactions among brain regions in a cuprizone model consistent with multiple sclerosis lesions**

**Supplemental figure**

**Figure S1. Expression of Mbp, Cd68 and Gfap at the spot level was similar to the IHC pattern in different treatment phenotypes.**

**Figure S2. Scatter plots depicting the consistency in cell type proportions predicted by cell2location in the same region of two distinct samples, both subjected to the same treatment.**

**Figure S3. NEBULA DE results for the snRNA-seq cell types in the CPZ vs CTL contrast.** The point color represents the FC direction with a significance cutoff of absolute (FC) > 1.5 and an adjusted p value < 0.05.

**Figure S4: Abundance of cell types and regional heterogeneity of gene expression depicted by spatial data.** (A) Spatial plot showing the abundance of selected cell types in samples from different treatments using cell2location. (B) Differential expression of astrocyte genes in the cortex compared to the CC across different treatments represented by a Venn diagram (top) and volcano plots (bottom). (C) Differential expression of OPC genes in the cortex compared to the CC across different treatments represented by a Venn diagram (top) and volcano plots (bottom).

**Figure S5. Visualization of SpaTalk receptor pathways in the CC and cortex.** The horizontal axis represents the section and condition of each replicate for which SpaTalk analysis was performed. The vertical axis on the left shows the enriched receptor pathway, the cell type sender-receiver pair for which the pathway was enriched, and the most significant LR pair that contributed to the enrichment. The vertical axis on the right shows the region in which the pathway was enriched. The point size represents the percentage of genes from a receptor pathway expressed in the sender receiver pairing. The point color represents the significance of the receptor pathway enrichment test. The point shape indicates the receptor pathway. Plotted pathway enrichments were required to be detected exclusively in one condition (CPZ, RCV, or CTL) within either the CC or cortex for a given sender–receiver pair and must be significant (p<0.05) within that region in at least 2 out of the 3 sections analyzed within that condition. The most significant LR interaction within a condition contributing to the pathway enrichment result is plotted in brackets beside the pathway name along the vertical axis. The figure shows the manual selection of a subset of pathway results that met these criteria.

**Figure S6. Full mapping of microglia and oligodendrocyte subclusters on Visium sections.**

 **Figure S7. Full DEG analysis of snRNA-seq dataset by cell type annotations.**

**Figure S8. Mean expression and the fraction of expressing nuclei for established markers of each of the 32 annotated cell types; all cell types displayed specific expression of their expected canonical markers, supporting the purity of the annotation.**

**Supplemental table title**

**Table S1. Significant sender–receiver cell pairs and enriched receptor pathways in the cortex predicted by SpaTalk.**

| **sender:receiver** | **receptor_pathways** |
| --- | --- |
| astrocytes:microglia | CRMPs in Sema3A signaling |
| astrocytes:microglia | Death Receptor Signalling |
| astrocytes:microglia | Herpes simplex virus 1 infection |
| astrocytes:microglia | Interleukin-12 family signaling |
| astrocytes:microglia | Interleukin-27 signaling |
| astrocytes:microglia | Late stage (branching morphogenesis) pancreatic bud precursor cells |
| astrocytes:microglia | MAP2K and MAPK activation |
| astrocytes:microglia | Pre-NOTCH Expression and Processing |
| astrocytes:microglia | Pre-NOTCH Processing in Golgi |
| astrocytes:microglia | Prion diseases |
| astrocytes:microglia | PTK6 promotes HIF1A stabilization |
| astrocytes:microglia | Regulation of Beta-Cell Development |
| astrocytes:microglia | Regulation of FZD by ubiquitination |
| astrocytes:microglia | Signaling by Non-Receptor Tyrosine Kinases |
| astrocytes:microglia | Signaling by NOTCH |
| astrocytes:microglia | Signaling by PTK6 |
| astrocytes:microglia | TNF signaling |
| astrocytes:microglia | TNFR1-induced NFkappaB signaling pathway |
| microglia:astrocytes | ECM-receptor interaction |
| microglia:mol | Disassembly of the destruction complex and recruitment of AXIN to the membrane |
| microglia:mol | Interleukin-12 family signaling |
| microglia:mol | Interleukin-27 signaling |
| microglia:mol | Interleukin-6 family signaling |
| microglia:mol | Interleukin-6 signaling |
| microglia:mol | Kaposi sarcoma-associated herpesvirus infection |
| microglia:mol | MAPK1 (ERK2) activation |
| microglia:mol | MAPK3 (ERK1) activation |
| microglia:mol | Parathyroid hormone synthesis, secretion and action |
| microglia:mol | RAF-independent MAPK1/3 activation |
| microglia:mol | TCF dependent signaling in response to WNT |
| microglia:mol | Wnt signaling pathway |
| microglia:opc | Growth hormone synthesis, secretion and action |
| microglia:opc | Human immunodeficiency virus 1 infection |
| microglia:opc | Interleukin-12 family signaling |
| microglia:opc | IRS activation |
| microglia:opc | Non-alcoholic fatty liver disease (NAFLD) |
| microglia:opc | PD-L1 expression and PD-1 checkpoint pathway in cancer |
| mol:microglia | Chemokine signaling pathway |
| mol:microglia | IRAK2 mediated activation of TAK1 complex upon TLR7/8 or 9 stimulation |
| mol:microglia | Regulation of FZD by ubiquitination |
| mol:microglia | TRAF6-mediated induction of TAK1 complex within TLR4 complex |
| mol:opc | Death Receptor Signalling |
| mol:opc | NOTCH3 Activation and Transmission of Signal to the Nucleus |
| mol:opc | p75 NTR receptor-mediated signalling |
| mol:opc | Signaling by NOTCH3 |
| opc:mol | Adherens junction |
| opc:mol | Cell adhesion molecules (CAMs) |
| opc:mol | ECM-receptor interaction |
| opc:mol | Signaling by MET |

**Table S2. Significant sender–receiver cell pairs and enriched receptor pathways in CC predicted by SpaTalk.**

| **snd_rec** | **receptor_pathways** |
| --- | --- |
| astrocytes:microglia | Endocrine resistance |
| astrocytes:microglia | Herpes simplex virus 1 infection |
| astrocytes:microglia | MAP2K and MAPK activation |
| astrocytes:microglia | Notch signaling pathway |
| astrocytes:microglia | Th1 and Th2 cell differentiation |
| microglia:astrocytes | Basal cell carcinoma |
| microglia:astrocytes | Melanogenesis |
| microglia:mol | Endocytosis |
| microglia:mol | JAK-STAT signaling pathway |
| microglia:opc | Bladder cancer |
| microglia:opc | Fibronectin matrix formation |
| microglia:opc | Signaling by Receptor Tyrosine Kinases |
| mol:microglia | Adipocytokine signaling pathway |
| mol:microglia | Amyotrophic lateral sclerosis (ALS) |
| mol:microglia | CRMPs in Sema3A signaling |
| mol:microglia | Endocrine resistance |
| mol:microglia | Noncanonical activation of NOTCH3 |
| mol:microglia | PTK6 promotes HIF1A stabilization |
| mol:microglia | Regulation of FZD by ubiquitination |
| mol:microglia | Signaling by Non-Receptor Tyrosine Kinases |
| mol:microglia | Signaling by PTK6 |
| mol:microglia | TNF signaling pathway |
| mol:microglia | TNFR2 non-canonical NF-kB pathway |
| mol:opc | Basal cell carcinoma |
| mol:opc | Cushing syndrome |
| mol:opc | RET signaling |
| opc:microglia | Endocrine resistance |
| opc:microglia | Fibronectin matrix formation |
| opc:microglia | Kaposi sarcoma-associated herpesvirus infection |
| opc:microglia | MAP2K and MAPK activation |
| opc:mol | Developmental Biology |

**Table S3. Full DEG results. (The table is provided as a separate excel file due to the large file size.)**
